# Effect of manual approaches with osteopathic modality on brain correlates of interoception: an fMRI study

**DOI:** 10.1038/s41598-020-60253-6

**Published:** 2020-02-21

**Authors:** Francesco Cerritelli, Piero Chiacchiaretta, Francesco Gambi, Mauro Gianni Perrucci, Giovanni Barassi, Christian Visciano, Rosa Grazia Bellomo, Raoul Saggini, Antonio Ferretti

**Affiliations:** 1 Department of Neuroscience, Imaging and Clinical Sciences, “G. D’Annunzio” University of Chieti-Pescara, Chieti, Italy; 2 ITAB-Institute for Advanced Biomedical Technologies, “G. D’Annunzio” University of Chieti-Pescara, Chieti, Italy; 3 Clinical-Based Human Research Department—C.O.M.E. Collaboration ONLUS, Pescara, Italy; 4 Department of Medical Oral and Biotechnological Science, “Gabriele d’Annunzio” University, Chieti-Pescara, Italy; 5 Department of Biomolecular Sciences, ‘Carlo Bo’ University, Urbino, Italy

**Keywords:** Attention, Perception

## Abstract

The present randomised placebo controlled trial explored the extent to which osteopathic manipulative treatment (OMT) affects brain activity, particularly the insula, during both an “interoceptive awareness” and “exteroceptive awareness” task in a sample of 32 right-handed adults with chronic Low Back Pain (CLBP) randomly assigned to either the OMT or sham group. Patients received 4 weekly sessions and fMRI was performed at enrolment (T0), immediately after the first session (T1) and at 1 month (T2). The results revealed that the OMT produced a distinct and specific reduction in BOLD response in specific brain areas related to interoception, i.e., bilateral insula, ACC, left striatum and rMFG. The observed trend across the three time points appears uncharacteristic. At T1, a marginal increase of the BOLD response was observed in all the above-mentioned areas except the rMFG, which showed a decrease in BOLD response. At T2, the response was the opposite: areas related to interoception (bilateral insula and ACC) as well as the rMFG and left striatum demonstrated significant decreased in BOLD response. The findings of this study provide an insight into the effects of manual therapies on brain activity and have implications for future research in the field.

## Introduction

The interaction between the sense of touch and the body is a well recognised process that takes place at different neural levels, with different effects and mechanisms of action. In the brain, the effect of this interaction produces different mental representations or experiences - also called feelings - of the body^1^. These include inputs from the physiological milieu of the body in terms of metabolic, structural and functional conditions at any given moment, a concept referred to as interoception^2^. Those feelings can be modulated by different stimuli and sources, including touch, ultimately modifying the perception of the internal and external world^3^.

Several neurobiological studies posed that the insula (INS) is a critical hub for multimodal interoceptive integration, involved in interoceptive processes, such as awareness of sensations from the body^4^, but also exteroceptive elements, such as perception of pain^5,6^, taste^7–10^, smell^11^, and touch^12^. In fact, external stimuli, i.e. pain, smell or taste, serve as body-mapped signals of the so-called peripersonal space [that is the space immediately surrounding the body]^13,14^. Moreover, in the anterior insula exteroceptive and interoceptive information overlap with emotional domains^11^, suggesting an underlying commonality^15^. In fact, the insula has been referred as a meeting point between external and internal milieus^16–19^. Detailed reviews of the interoceptive evidence are available elsewhere^2,3,20^.

Methods to measure interoceptive differences comprise the use of questionnaires and behavioural tests that either exploit natural fluctuations in internal physiological signals or manipulate organ physiology experimentally^21,22^. For practical reasons, heartbeat detection tasks are measures largely utilised to assess differences among individuals regarding interoceptive accuracy and ability^23–25^. These tests quantify an individual’s ability to distinguish his own heartbeat at rest, by counting, tapping or by judging heartbeat timing relative to an external stimulus. Due to their nature, these tasks can be utilized in the context of an fMRI study with good validity and reliability^26^.

Low back pain (LBP) is a widespread health problem and a major cause of disability worldwide^27,28^. Lifetime prevalence estimates of LBP range from 60% to 70%^27^. In Europe, LBP represents the second highest cause of morbidity measured by disability-adjusted life years^28^. Research evidence suggests that several brain areas are modified after exposure to chronic low back pain (CLBP) - typically defined as pain lasting more than 3 months^29^. Indeed, these changes seem to be correlated to changes in the anterior INS and Anterior Cingulate Cortex (ACC)^30^. Other studies highlighted the predominance of the medial prefrontal cortex (mPFC) as a key regulator of pain perception^31^. Apkarian *et al*. demonstrated that modelling the activity of mPFC and INS it is possible to quantify the magnitude and duration of back pain with an error of 20%^31^. Furthermore, other research showed that subjects with chronic pain are characterised by an abnormal function of the resting state networks with higher firing on the INS and ACC^32–34^, as well the mPFC^33^. Taken together, these studies seem to indicate a critical role for the INS and ACC in the modulation of pain.

Preliminary research in field of manual therapies such as osteopathy indicates that interoception mediated through the sense of touch may play an important role in their therapeutic effects^35–37^. Touch is recognised as both an exteroceptive and interoceptive modality, where the latter seems to be supplied by small-diameter low-conducting unmyelinated C-tactile (CT) fibres, which uses the lamina I spinothalamocortical tract to subserve homeostasis and create the basis for feelings^2^. Researchers have proposed an ‘affective’ homunculus in the insula that maps the hedonic properties of gentle touch, based on the inferred increase in innervation density of CTs in more proximal body sites^38^. On this point, the CT fibres might be key neurobiological component in the touch-based manual therapies underpinning mechanisms^38^.

Although not formally tested, it can be argued that the link between the observed manual therapy effects, specifically osteopathy, and interoception is plausible, particularly in the light of current neuroscience literature. Our hypothesis, therefore, is that touch-based therapy can affect neural activity when perceiving heartbeat. In this study, we investigated the effect of an osteopathic treatment on brain correlates, specifically insula-based networks, on patients with CLBP.

## Results

### Description of the sample at baseline

Thirty-two right-handed patients were randomised and divided into the study (N = 16) and control groups (N = 16). Three patients (1 in the study group and 2 in the control group) dropped out during the trial, leading to a final sample size of 29 patients. At baseline, there were no statistically significant differences between groups in terms of age, gender, BMI, marital situation, and academic and professional qualifications (Table 1). Considering the pain measurements, groups were comparable for level of pain measured by VAS and McGill score, and disability index measured both by Roland-Morris and Oswestry (Table 1).

**Table 1:** Description of the sample at the baseline.

	Study group (OMT)	Control group (Sham)	p < |t|
Age	41.8 ± 6.6	42.7 ± 8.0	0.73
Gender			0.70*
Male	9 (60)	11 (73.3)	
Female	6 (40)	4 (26.7)	
BMI	24.1 ± 3.5	25.5 ± 2.4	0.19
Civil condition			1.00*
Unmarried	7 (46.7)	6 (40)	
Married	8 (53.3)	9 (60)	
Study title			0.62*
Middle school	2 (13.3)	2 (13.3)	
High school	4 (26.7)	5 (33.4)	
Bachelor degree	5 (33.3)	2 (13.3)	
Master degree	4 (26.7)	6 (40)	
Disease duration (m)	15.1 ± 9.2	14.1 ± 6.7	0.72
**General scores**			
BAQ	68.5 ± 31.4	51.8 ± 30.0	0.15
STAY-Y1	42.4 ± 3.4	42.7 ± 2.9	0.85
STAY-Y2	41.3 ± 3.0	41.1 ± 3.7	0.87
**Pain scores**			
McGill			
S-PRI	16.5 ± 5.0	16.6 ± 5.9	0.94
A-PRI	6.3 ± 2.7	6.3 ± 2.7	1.00
T-PRI	22.7 ± 6.8	22.9 ± 8.1	0.96
PPI	3.5 ± 0.5	3.5 ± 0.6	1.00
VAS	63.1 ± 21.4	57.5 ± 17.3	0.1
Oswestry	24.9 ± 3.3	26.0 ± 5.2	0.51
Roland-Morris	15.5 ± 4.0	15.3 ± 4.9	0.9

P values from t test. *p values from X^2^ BMI = Body Mass Index; BAQ = Body Awareness Questionnaire; S-PRI = Sensory Pain Rating Index; A-PRI = Affective Pain Rating Index; T-PRI = Total Pain Rating Index; PPI = Present Pain Intensity Index; VAS = Visual Analogue Scale.

### Behavioural results

When the mean error for the IA is considered, the results of MVM analysis showed a group × time interaction effect (F = 6.78; p < 0.01) but not group effect (F = 4.98; p = 0.12).

**Figure 1:**
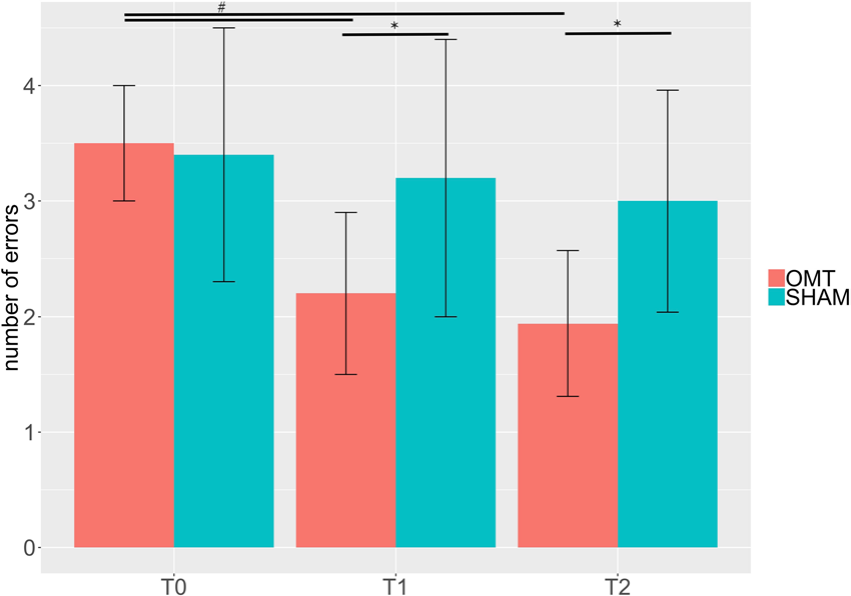
Bar chart of behavioural data. Data show the mean errors in the Interoceptive Attentive task (Mean ± SD). ^*^Statistically significant values between groups. ^#^Paired t test statistically significant values within OMT group.

Indeed, no statistically differences were observed between groups at both baseline (OMT: 3.5 ± 0.50 vs sham 3.4 ± 1.1; t = 0.33, df = 20.95, P = 0.74, two-tailed) (Fig. 1). Statistically significant differences were found at T1 (OMT: 2.2 ± 0.7 vs sham 3.2 ± 1.2; t = −2.88, df = 24.15, p-value <0.001) and T2 (OMT: 1.94 ± 0.63 vs sham 3.0 ± 0.96; t = −3.69, df = 25.90, p-value <0.001). Besides, OMT group showed a significant reduction compared to baseline both at T1 (mean of differences: 1.30; 95% CI: 0.86–1.74; t = 6.04; df = 27.15; p < 0.001) and T2 (mean of differences: 1.53; 95% CI: 1.15–1.97; t = 7.76, df = 28.53, p-value <0.001). Analysis of the EA task did not reveal any differences between groups and time. Amongst 14 patients allocated to the sham group, none of them was able to correctly guess the nature of the treatment.

### fMRI results

To study the relationship between IA, OMT and brain activity in the insula and in other correlated interoceptive areas, a well-established fMRI paradigm^39^ including IA and EA tasks, was applied. Task difficulty effects between interoceptive and exteroceptive awareness (IA and EA) were excluded by showing no significant differences between the total mean error of IA (mean: 3.43 ± 0.6) and EA (mean: 2.28 ± 0.53) condition (t = 1.43, df = 470.63, p-value = 0.15).

**Figure 2:**
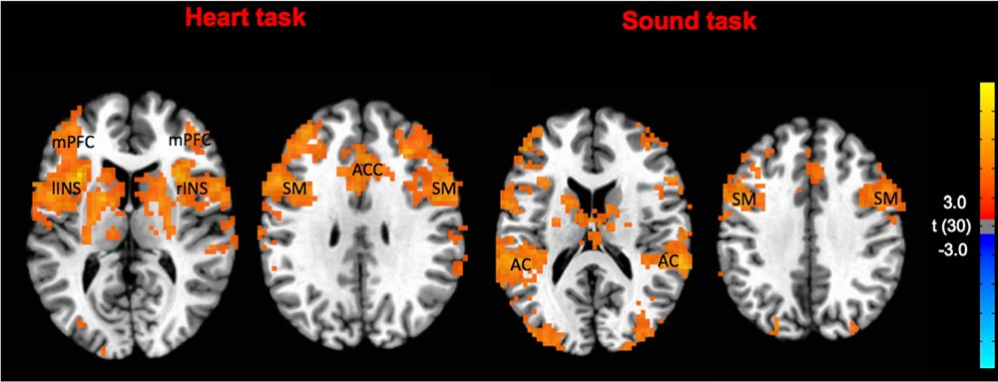
Task specific brain activations at T0 pooling the two groups. The group statistical maps were thresholded at p < 0.05. mPFC: medial prefrontal cortex; lINS: left insula; rINS: right insula; SM: somatomotor cortex; ACC: anterior cingulate cortex; AC: acoustic cortex.

**Figure 3:**
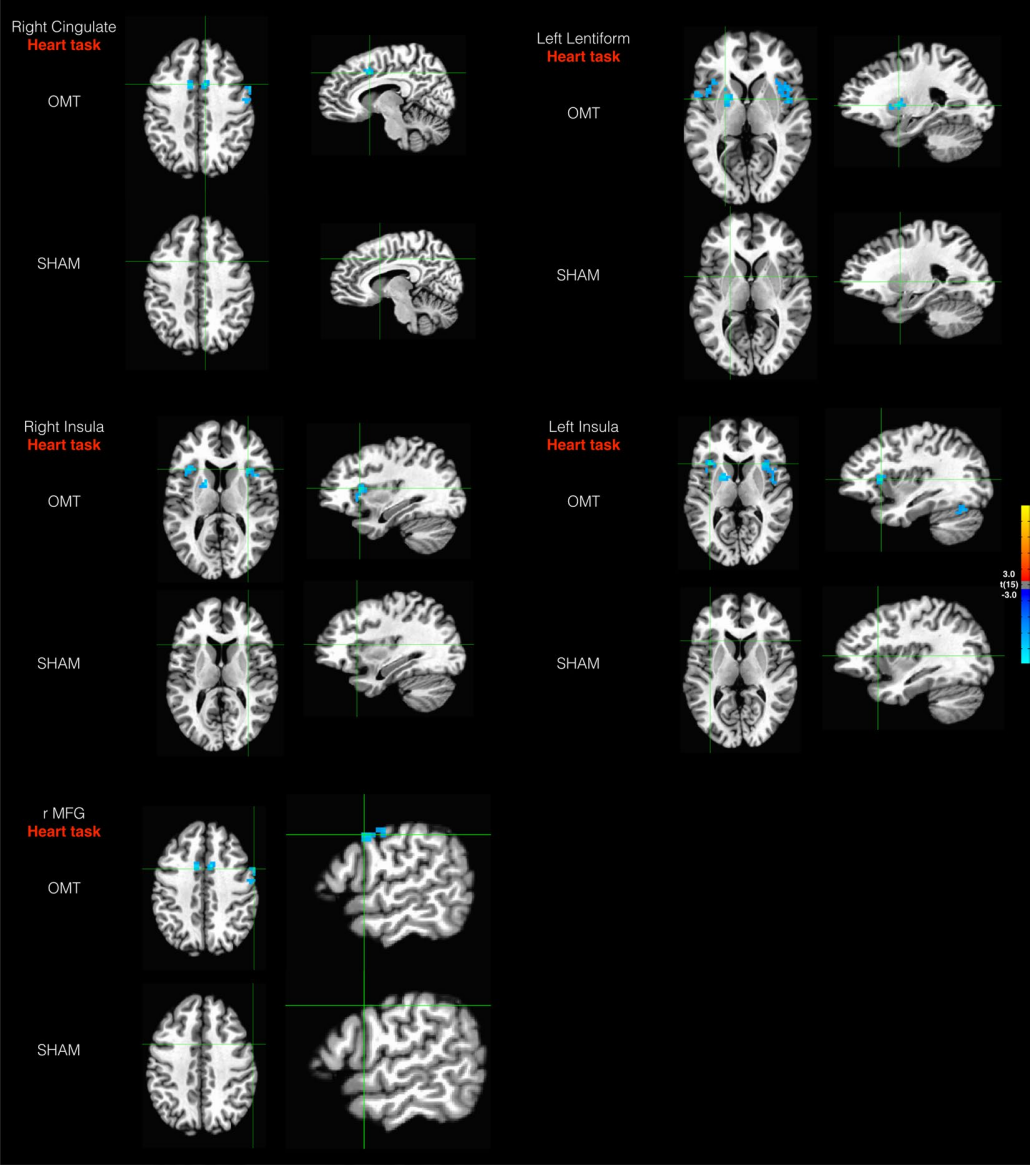
Results of the group analysis revealing areas for OMT and Sham group by the contrast T2-T1-T0 in the heart task (interoceptive awareness task) and sound task (exteroceptive awareness task). The group statistical maps were thresholded at p < 0.05, corrected for multiple comparisons using FDR, and superimposed on the Talairach transformed structural scan of one of the subjects. Activation was observed in Right Cingulate Cortex, Right and Left Insula, Left Lentiform and right Middle Frontal Gyrus (rMFG).

Firstly, the group activation maps at T0 showed a significant activation of bilateral insula, bilateral cingulate cortex, bilateral sensory-motor cortex and medial prefrontal cortex for the IA task and bilateral superior temporal gyrus, bilateral sensory-motor cortex for the EA task (Fig. 2). At baseline, there were no differences between OMT and SHAM group in the activation of interoceptive and exteroceptive areas. These results confirm that the two groups were balanced regarding the interoceptive and exteroceptive tasks. Considering ROIs analysis for the IA task, there was a significant effect of group (OMT < SHAM) in the right anterior cingulate cortex (ACC, F = 10.81; p < 0.001), right insula cortex (rINS, F = 10.12; p < 0.001), left insula cortex (lINS, F = 9.96; p < 0.001), left striatum (F = 11.42; p < 0.001) and right middle frontal gyrus (rMFG, F = 7.12; p < 0.001) (Fig. 3). Significant effects were found also for time (ACC: F = 8.67, p < 0.001; rINS: F = 8.98, p < 0.001; lINS: F = 9.11, p < 0.001; left striatum: F = 7.67, p < 0.001; rMFG: F = 8.42, p < 0.001) as well as group × time interaction (ACC: F = 10.21, p < 0.001; rINS: F = 10.43, p < 0.001; lINS: F = 10.01, p < 0.001; left striatum: F = 11.67, p < 0.001; rMFG: F = 10.11, p < 0.001).

**Figure 4:**
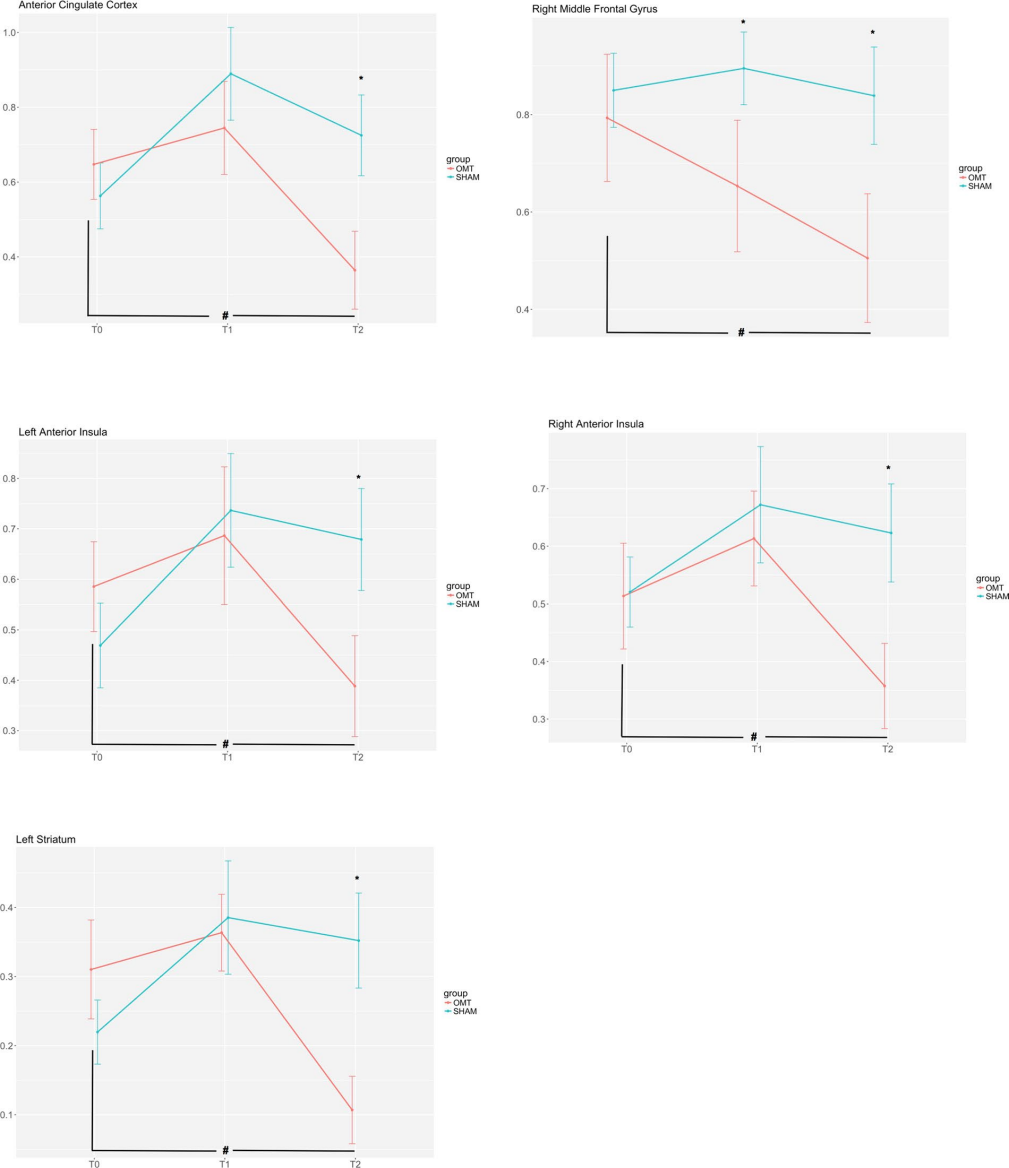
Activation (GLM beta values ± SEM) over time for the two groups and the different regions of interest. ^∗^Statistically significant values between groups after Bonferroni-Holm correction. ^#^Paired t test statistically significant values within OMT group.

Comparing the two groups, OMT showed a significant reduction of beta values in the ACC (t = −4.07, p < 0.001), rINS (t = −3.87, p < 0.001), lINS (t = −3.16, p < 0.001), left striatum (t = −4.97, p < 0.001) and rMFG (t = −3.45, p < 0.001) mainly at T2 (Fig. 4). Interestingly, only the rMFG revealed a statistically significant different between the two groups at T1 (t = −2.65, p < 0.01).

The within group timepoints comparison showed statistically significant differences for the OMT group only. In general, there was a significant difference between T2 and T0 beta values for ACC (T2 vs T0: t = −2.97, p < 0.01), lINS (T2 vs T0: t = −2.47, p < 0.01), rINS (T2 vs T0: t = −2.35, p < 0.01), left striatum (T2 vs T0: t = −3.34, p < 0.01) and rMFG (T2 vs T0: t = −2.54, p < 0.01).

In relation to the EA task, the OMT and sham groups were comparable, with no significant difference detected in brain activation.

## Discussion

The present study showed that osteopathic manipulative treatment produces a distinct and specific BOLD response in specific areas related to interoception. Compared to the sham group, patients receiving OMT demonstrate these effects specifically in the rINS, lINS, ACC, left striatum and rMFG. The trend across the 3-time points seems to be uncharacteristic. Indeed, immediately after the first OMT session, it was shown a marginal increase in BOLD response in all the above-mentioned areas but not for the rMFG, which showed a decrease. At T2, the response appeared to be opposite: areas related to interoception (bilateral insula and ACC) as well as rMFG and left striatum significantly decreased the BOLD response, i.e. these clusters exhibited a clear modulation after 4 OMT sessions. This is particularly observable in the OMT group when compared to the other time points (T0 and T1) and to the sham group.

Besides, considering the mean errors in the IA task, results showed that at T2 the OMT group reduced the number of errors compared to baseline and compared to the sham group. This might suggest that participants in the OMT group improved their ability in the heartbeat tracking task, a measure of interoceptive accuracy. It is noteworthy to consider that the chance of task habituation and thus predictability of the heartbeat tracking was controlled by the randomisation of the task (both between IA-EA and within the task sequence). In addition, it seems to be unlikely that patients performing the IA task at baseline, that means 4 blocks by 15 sec each (one minute in total), would have been adapted and trained for that task and thus influence the performance one month later. This is confirmed by the fact that mean errors in the sham group were similar between T0 and T2. Considering the above-mentioned points, it might be possible that the use of OMT procedures could improve the perception of heartbeat, enabling patients to detect more accurately their own heartbeat. It is well-established in the literature that to perceive your own heartbeat the salience network needs to be recruited^26,40,41^. It can be argued that a more efficient network means a better ability to perform the IA task and thus feel more accurately one’s own heartbeat^42^. Thus, we could speculate that the use of OMT might produce specific effects in interoceptive brain areas, possibly reflecting an increased efficiency to decode bottom-up interoceptive heart-based stimuli.

In addition to the salience areas activated, the rMFG activation seems to show a similar pattern. Interestingly, under the Corbetta’s “circuit-breaker” proposal^43^, the right MFG would be in charge of the modulation of exogenous and endogenous attention. Japee and colleagues have revealed that the rMFG could play an important role in reorienting attention from exogenous to endogenous attentional control^44^. Considering the findings of the current study, we would argue that the use of OMT can influence activity in the rMFG allowing a more precise attentional control in order to re-orient more efficiently one’s own attention towards endogenous stimuli. Although not formally tested yet, we can hypothesise that the use of OMT might act on the rMFG facilitating the switch from external to internal milieu. This would eventually impact on the accuracy of perception from within the body.

This study has some limitations that should be outlined. Although the analysis presented here was robustly driven by a specific rationale, it can be considered a first step that will be complemented by further work based on more complex approaches to assess e.g., functional and effective connectivity during both resting and task paradigms. For example, graph-theory analysis (GTA)^45,46^, can yield metrics for both connectivity profiles and network efficiency, enabling researchers to examine the efficiency of individual nodes to integrate signals at global and local levels. Previous research based on GTA has demonstrated that brain network is structured in a ‘small-world’ topology characterised by dense intra-modular connections and relatively few inter-modular connections^45–47^. In the context of the present study, GTA might be useful to test any distinct brain networks during post-osteopathic resting state. This will provide direct evidence that the post-treatment resting state contains osteopathic-related effects that might be due to the specific touch-based nature of osteopathy.

Furthermore, effective functional connectivity (defined as the influence that one area exerts on another) during tasks can be investigated using Dynamic Causal Modeling^48^. This will lead us to investigate interactions among relevant regions involved in the exteroception/proprioception vs interoception by analysing the effective connectivity between brain regions.

The study of manual therapies from a neuroscience point of view might bring new insights within a still unexplored research field. Despite the large use of different manual approaches and touch-based interventions^49^, research on brain activity is still lacking. Neuroscience can provide different methodologies allowing researchers to decode unique patterns within- and between-manual treatments. In addition to this, setting up ecologically-relevant studies is essential. On this point, selecting appropriate samples is a prerequisite as well as building up robust rationale is necessary. Too often, studies in the context of manual therapies lack of details, significantly impairing the validity, reliability and clinical applicability of the research.

The present research investigated the effect of OMT on brain correlates, specifically insula-based networks, in patients with CLBP. The findings of the study, particularly the observed changes in the insular cortex and its associated interoceptive role, support the hypothesis previously proposed by D’Alessandro and co-workers that manual therapy might exploit an interoceptive paradigm which may explain some of the clinical effects of manual treatments^35^. Therefore, we would argue that the present research with preliminary clinically-based evidence, can provide an insight into the effects of manual therapies on brain activity and have implications for future research in the field.

## Methods

The present randomised placebo-controlled trial was designed to explore the extent to which OMT can change activity in the insula and therefore of its anterior circuit during both an “interoceptive awareness” and “exteroceptive awareness” task in a sample of CLBP patients.

### Population

Patients were recruited from the outpatient department at a neurological and orthopaedic rehabilitation centre of the University of Chieti (CUMFER). Interventions, assessments and data collection, and data analysis were conducted at the same study site. Adult patients (≥18 years and ≤60 years old) referred to the trial clinic for Chronic Non-specific Low Back Pain (CLBP) treatment by their general practitioner or specialist were invited to participate in the trial. If no medical referral was given, e.g. as a response to the public invitation in local print media, an independent orthopaedic specialist at the study site examined the patient for eligibility and to confirm diagnosis (CLBP). Symptoms included any chronic (>3 months) pain or discomfort localised below the costal margin and above the inferior gluteal folds, with or without referred leg pain^50^. Written, informed consent was provided prior to the beginning of any of the study procedures.

Meeting any of the following criteria led to exclusion^51^: clinical sign of neurological damage with sensorimotor impairments (i.e., radicular syndrome, paresis or tingling in limbs); suspected or confirmed spinal pathology (e.g., tumour, infection, fracture or inflammatory disease); history of spinal surgery (e.g., decompensation or stiffening); whiplash injury within the last 12 months; cervical pain that reduces active movement to less than 30° rotation to each side; known vestibular pathologies; major surgery scheduled during study period; physiotherapy during the last 12 weeks; inability to follow the procedures of the study - e.g., due to language problems, psychological/psychiatric disorders, dementia and parallel participation in another study. At enrolment, eligible patients were assessed by a senior medical doctor in order to confirm the diagnosis and to exclude psychiatric disease and/or any other exclusion criteria.

The procedures were approved by the local ethics committee (University of Chieti-Pescara number: 7/09-04-15) and conform to the Declaration of Helsinki. The protocol (supplementary materials provide details of the protocol) was registered on clinicaltrial.gov (ID: NCT02464475 on 08/06/2015). No data was recorded before written informed consent to participate and to publish was given by the participant.

### Randomization and masking

Eligible patients were randomly divided into two groups using a 1:1 ratio and were assigned to either the OMT group or Sham group. Block randomization was applied according to a computer-generated randomization list using a block size of 10. All patients, allocated using sealed envelopes, were not aware of any step of the study design as well as outcomes or group allocation. The randomization list was stored in a dedicated and protected web-based space and an information technology consultant was in charge for the entire process. Research staff were kept blinded to the randomisation list and to patient allocation throughout the study. Moreover, they were blinded to patients’ allocation, since all patients had a touch-based intervention by the practitioner. Only the osteopathic practitioner was aware of the patients’ group allocation. Moreover, the practitioner who performed OMT had no role in patient care decisions. The researchers operating the fMRI and dealing with fMRI data were unaware of patients’ allocation.

### Prescan behavioural assessment

At T0, before the fMRI scan, patients were asked to complete paper-based questionnaires. A socio-demographic questionnaire was administered to collect baseline data in terms of gender, BMI, age, academic degree, civil state, smoking habits and type of work. Besides, the Edinburgh Handedness Inventory was used to investigate the hand dominance (Oldfield, 1971) and the State-Trait Anxiety Inventory (STAI-Y1 and Y2) to test trait anxiety^52^.

The Body Awareness Questionnaire (BAQ) is considered a reliable and valid instrument for measuring self-reported attentiveness to bodily processes^53,54^. It is made of 18 statements that measure beliefs about one’s sensitivity to normal (i.e., non-emotive and non-pathological) bodily functions and the ability to anticipate bodily reactions. Items are answered on a seven-point Likert scale. Cronbach’s alpha coefficient for the BAQ was 0.80 for the Italian sample.

### Prescan pain assessment

Several tools were specifically used to assess pain perception in patients. The Roland–Morris Disability Questionnaire is a health status measure to assess physical disability low back pain patient. The questionnaire is composed by 24 yes/no items and has good psychometric properties, evidenced by internal consistency and responsiveness^55^. The Oswestry Low Back Disability Questionnaire (OSW) explores the disability derived from low back pain. The questionnaire consists of 10 items addressing different aspects of functioning (e.g., pain intensity, physical functioning, sleep functioning, social functioning). The reliability, discriminant and construct validities of the OSW are good^56^. The McGill Pain Questionnaire (MPQ) is a widely used tool to assess pain features, with reference to its sensory and affective qualities^57,58^. The MPQ is composed by 15 items describing the pain sensation (11 sensory and 4 affective) which are self-rated by the patient according to their intensity level on a 0–3 Likert scale. The reliability and validity of these measures are good and well-documented^59^.

### Experimental design and description of the paradigm

All eligible patients were randomised in a study group (OMT) and control group (Sham). The study group underwent four weekly sessions of OMT of 30 minutes each. Osteopathy is a nonpharmacological, non-invasive manual medicine, regarded by some as Complementary and Alternative Medicine (CAM). A series of manual techniques are applied by osteopathic therapists to improve bodily function altered by any somatic (body framework) dysfunction (ICD- 10 code: M99.0–99.9)^60^. In the osteopathic practice two are the essential elements: a structural evaluation for diagnosis and a pool of different manipulative techniques for the treatment. The aim of the structural assessment is to identify specific somatic dysfunctions. Diagnostic criteria for somatic dysfunctions focus on the tone and possible abnormalities of tissue texture. Areas of asymmetry and misalignment of bony landmarks are also evaluated, along with the quality of motion, balance, and organization. The term OMT currently includes>20 types of manual treatments administered by osteopaths^61^.

In the current study the treatment was administered by a registered and licensed osteopath. The techniques used for the current study were: balanced-ligamentous tension, balanced-membranous and fluidic techniques, in line with the principles and procedures available in the current osteopathic literature. In brief, these techniques are classified as indirect approaches^62^ and use light, gentle touch^38^ to correct the somatic dysfunction by applying the Sutherland’s point of maximum freedom (balance point) model^62^. All treatment sessions took place in the CUMFER.

The control group received a sham treatment, i.e., sessions without applying any type of osteopathic technique or procedure. Specifically, the operator performed an osteopathic-like manual assessment without paying attention to bodily areas with somatic dysfunctions. After the evaluation, the operator asked the patient to lay down on the plinth and gently placed the hands on a pre-defined number of anatomical areas without applying any type of technique but just using a gentle static or dynamic touch. The parts identified in the protocol were: lumbar spine, sacrum, pelvis, diaphragm, upper thorax, cervical spine and cranium. The sequence to apply during the session was decided by the operator before the session. This was planned to prevent any possible chance from the patient to guess the group allocation. The sessions lasted 30 minutes, as for the OMT, took place in the same location/room and were administered by the same practitioner. This to avoid any possible contamination and allocation bias.

During the study period, all patients were asked to avoid if possible any form of medication.

**Figure 5:**
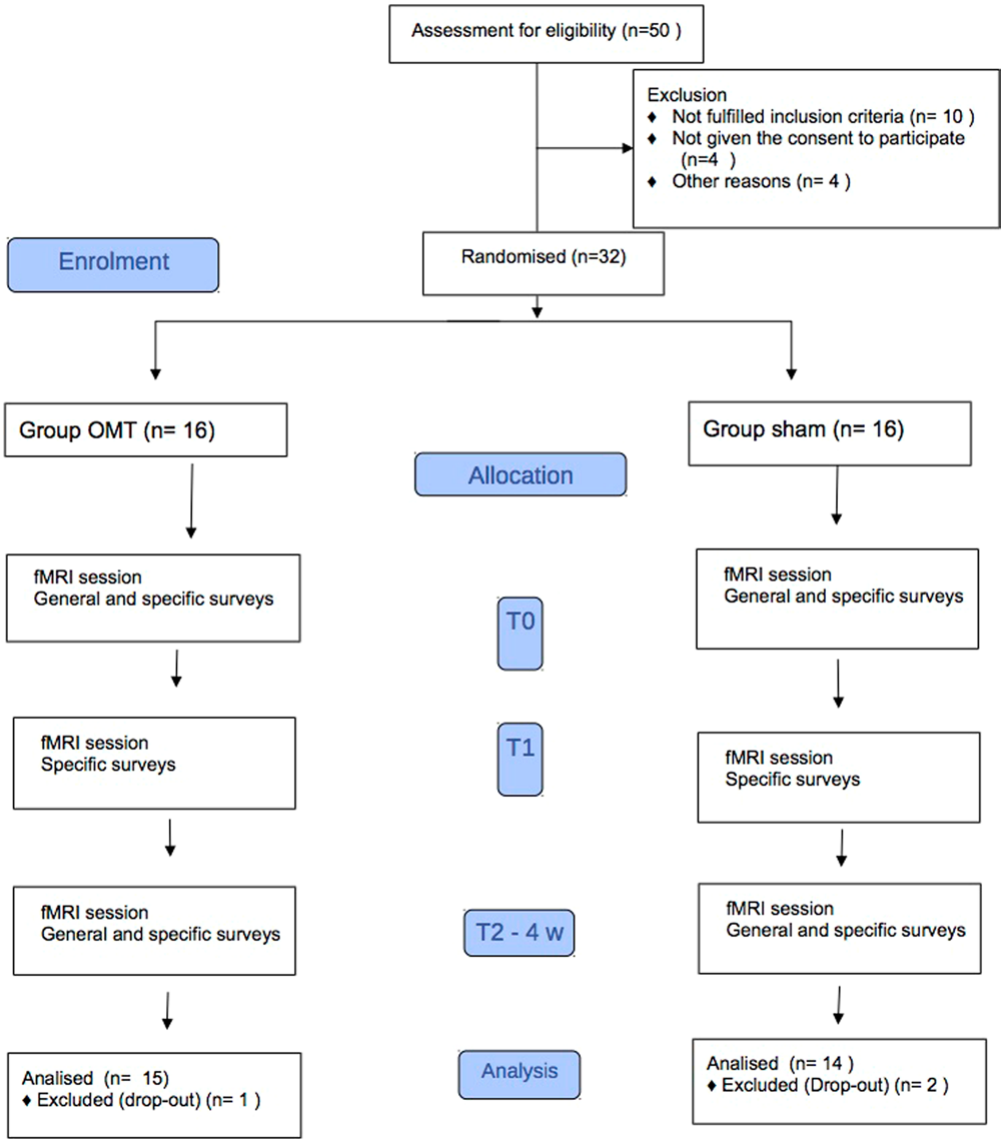
Flowchart of the study.

The study period was organised as follows with fMRI sessions at three major time points (Fig. 5):

1. Baseline (T0): before the treatment.
2. Acute response (T1): Immediately after the first manual session.
3. Chronic response (T2): at the end of the study period (after a month), which included four treatment sessions.

After the clinical evaluation (enrolment) and at T2, patients were asked to fill in the paper-based questionnaires.

### Description of the paradigm

The fMRI design used to assess brain correlates of interoceptive and exteroceptive awareness was derived by previous research where it was successfully tested^39,63–65^. Specifically, we used a block design with 3 conditions:

- heartbeat tracking for interoceptive awareness (IA).
- auditive tracking for exteroceptive awareness (EA).
- resting period (fixation period) where no structured thinking or action was required to subjects.

**Figure 6:**
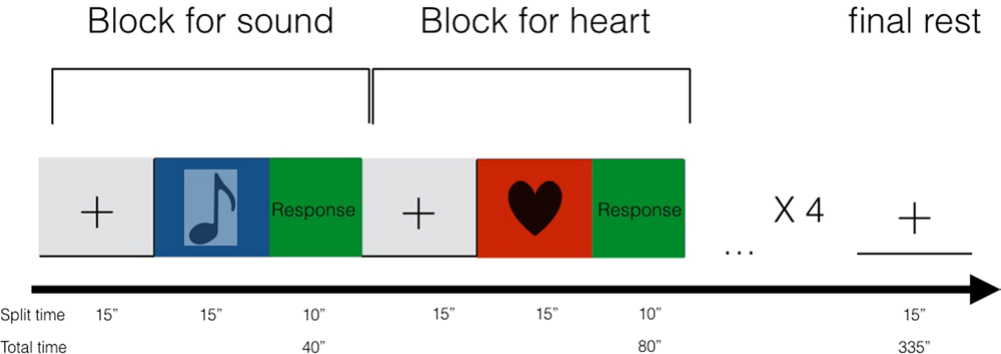
Description of the fMRI paradigm.

In order to limit cognitive processes other than intero- or exteroception, simple visual stimuli were used to indicate the task type. These visual cues were projected via an LCD projector onto a screen visible through a mirror mounted on the headcoil and consisted of symbols described as follows (Fig. 6):

1. Heart, i.e. heartbeat tracking for interoceptive awareness – IA.
2. Treble clef, i.e. auditive tracking for exteroceptive awareness – EA.
3. Dark cross, i.e. resting period (fixation period) where no structured thinking or action was required to subjects.

For task 1, a dark coloured heart on a light background was presented. As long as this cue was visible on the screen, participants were asked to silently count their own heartbeat (a modified version of the Schandry’s original heartbeat tracking task^66^). Subjects were instructed to breathe normally and any form of helping strategies (i.e. taking the pulse rate) were not allowed. Furthermore, participants were boosted to consider only heartbeats they were convinced but also advised to recognise weak feelings.

For task 2, the symbol of a dark coloured treble clef with the same size as the heart symbol was presented on the same light background. During this task individuals silently counted the number of tones played through fMRI compatible headphones (NordicNeuroLab audio system).

Each cue was showed on the screen in blocks with a duration of 15 seconds during which the subject had to perform the related task. Four blocks for each task were alternated pseudorandomly with rest periods.

At the end of each task block, the patient had to report the number of heartbeats or tones counted using an fMRI compatible mouse with two buttons. The 2 buttons, left and right, were used to control the units and decimals, allowing the subjects to quickly report their evaluation. Specifically, the left button served to move from decimals to units and back, while the right button was used to select the corresponding quantities. The time for declaring the score was 10 seconds. Fixation periods were indicated by a dark cross (of the same size and colour as the IA and EA symbols) on a light background. Participants were instructed to relax and minimise any cognitive work during these periods. To avoid any habituation effect, the volume of the musical tone was adjusted before the beginning of the fMRI session while the subject was lying down. The total duration of the run was 335 seconds. The run was repeated twice, and the order of the stimuli changed randomly.

The delivery of visual cues was controlled by a software written in Matlab. Visual stimuli were projected via an LCD projector onto a screen visible through a mirror mounted on the headcoil. Immediately before the scan, participants had a practice session and were carefully instructed and familiarized with the task.

### fMRI data acquisition

Imaging was performed using a Philips Achieva 3 Tesla scanner (Philips Medical Systems, Best, Netherlands) equipped with an 8-channel phased-array head coil for signal reception and a whole-body radiofrequency coil for signal. First, high resolution structural volume was obtained using a 3D fast field echo T1-weighted sequence (sagittal, matrix 256 × 256, FOV = 256 mm, slice thickness = 1 mm, no gap, in-plane voxel size = 1 × 1 mm, flip angle = 12°, TR = 9.7 ms and TE = 4 ms). Then, Blood Oxygen Level Dependent (BOLD) fMRI data were acquired using a gradient-echo T2∗-weighted echo-planar (EPI) sequence (matrix 80 × 80, voxel size 3 mm × 3 mm × 3.5 mm, SENSE 1.8, TE = 30 ms, TR = 1.8 s, 185 volumes per run). During fMRI, physiological signals were recorded at 100 Hz using a pulse oximeter placed on a finger of the left hand and a pneumatic belt strapped around the upper abdomen. All the data were stored and secured at the Department of Neuroscience of University of Chieti-Pescara.

### fMRI data pre-processing

Analysis of fMRI data was performed using AFNI. Due to T1 saturation effects, the first five volumes of each run were discarded from the analysis. During preprocessing, transient signal spikes were removed from the EPI time series AFNI’s “3dDespike” and slice scan time correction was performed. Motion correction was done by aligning EPI images to the sixth volume of the first run. Then, preprocessed functional scans were coregistered with the corresponding structural data set, normalized to the MNI space, spatial smoothed (6 mm FWHM) and high-pass filtered (cut-off 0.013 Hz).

### Statistical analysis

#### Sample size calculation

The fMRI literature (e.g. comparison of connectivity values between different populations as in Greicius *et al*.^67^) reported expected effect size estimates to be relatively high (d of Cohen = 1.01). This effect-size, together with an alfa value of 0.05 and a Beta of 0.80 typical in neuroimaging studies^68^ have been included in the R statistical program to estimate the sample size, obtaining N = 16 subjects per group, values compatible with those proposed in this study.

#### Behavioural data

Arithmetic mean and standard deviation as well as median, percentage and range were used to explore the general characteristics of the study population. To compare the OMT and Sham group at enrolment, univariate statistical tests, student *t* test and chi square test were performed. To study the independent effect of OMT on primary and secondary endpoints, a repeated measure analysis based on linear mixed effect model was applied considering group differences (OMT vs Sham) across time (baseline vs. experimental timepoints) and conditions (Interoceptive vs Exteroceptive). To indicate statistical difference, two-tailed P values of less than 0.05 was considered. The significance threshold was further adjusted for multiple comparisons using Bonferroni’s correction. This data analysis was carried out using the R statistical program (v. 3.5.2). No adverse events were reported by any of the included patients.

#### fMRI data

First, statistical activation maps were obtained for individual subjects using the general linear model (GLM), considering the heart and sound conditions as predictors of interest, whereas, the periods corresponding to the subject’s response for both heart and sound were included in the model as predictors of no interest. A two-gamma standard hemodynamic response function was used in order to account for the hemodynamic delay. This analysis was performed in order to identify brain areas of increased/decreased BOLD signal while the subject was performing heart (IA) and sound (EA) tasks. Then, the individual beta values corresponding to the two tasks were used as input in a group analysis based on the multivariate modelling approach (MVM) as implemented in AFNI (program 3dMVM), to assess treatment effects on brain activation. The MVM approach offers increased flexibility with respect to traditional AN(C)OVA and GLM, with voxel level correction schemes when the sphericity assumption is violated^69^.

In particular the model included two groups (OMT vs Sham), 3-time points (T0, T1, T2) and 2 conditions (IA vs EA). Thus, it was a 2 × 3 × 2 factorial design where group was considered a between-group variable, whereas time and task were used as within-group factors.

First, the main contrasts T0_IA and T0_EA vs rest (pooling the two groups) were performed to search for areas activated at the group level by IA and EA tasks. The between-groups OMT_T0_IA vs Sham_T0_IA and OMT_T0_EA vs Sham_T0_EA contrasts were also performed, to check that the two groups were balanced regarding the level of activation during the two tasks at T0. Then, the contrasts OMT_(T2-T1-T0)_IA vs Sham_(T2-T1-T0)_IA and OMT_(T2-T1-T0)_EA vs Sham_(T2-T1-T0)_EA were performed to search for areas showing a significant effect.

Statistical maps obtained from these contrasts were thresholded at p < 0.05, corrected for multiple comparisons. Correction for multiple comparisons was performed using false discovery rate (FDR). Finally, to quantify activation changes across groups, time and tasks, a region of interest (ROI) analysis was performed. To avoid double dipping problems^70^, ROIs were defined using independent coordinates from the literature^71–76^. Spherical ROIs with a 9 mm radius were considered for the following areas: left insula (−36,20,4), right insula (32,18,4), cingulate cortex (8,16,36), striatum (−24,0,8), right middle frontal gyrus (56,8,38).

## Supplementary information

CONSORT Checklist.

Research protocol.

## References

[1] Damasio A, Carvalho GB (2013). The nature of feelings: evolutionary and neurobiological origins. Nat. Rev. Neurosci..

[2] Craig AD (2002). How do you feel? Interoception: the sense of the physiological condition of the body. Nat. Rev. Neurosci..

[3] Craig AD (2009). How do you feel–now? The anterior insula and human awareness. Nat. Rev. Neurosci..

[4] Khalsa SS, Rudrauf D, Feinstein JS, Tranel D (2009). The pathways of interoceptive awareness. Nat. Neurosci..

[5] Brooks JC, Nurmikko TJ, Bimson WE, Singh KD, Roberts N (2002). fMRI of thermal pain: effects of stimulus laterality and attention. Neuroimage.

[6] Gramsch C (2014). Learning pain-related fear: neural mechanisms mediating rapid differential conditioning, extinction and reinstatement processes in human visceral pain. Neurobiol. Learn. Mem..

[7] Gagnon L (2014). Neural correlates of taste perception in congenital olfactory impairment. Neuropsychologia.

[8] Iannilli E, Noennig N, Hummel T, Schoenfeld AM (2014). Spatio-temporal correlates of taste processing in the human primary gustatory cortex. Neurosci..

[9] Parabucki A, Netser S (2014). Origin of palatability coding in medial prefrontal cortex. J. Neurosci..

[10] van den Bosch I (2014). To like or not to like: neural substrates of subjective flavor preferences. Behav. Brain Res..

[11] Kurth F, Zilles K, Fox PT, Laird AR, Eickhoff SB (2010). A link between the systems: functional differentiation and integration within the human insula revealed by meta-analysis. Brain Struct. Funct..

[12] McGlone F, Wessberg J, Olausson H (2014). Discriminative and affective touch: sensing and feeling. Neuron.

[13] Couto B (2015). Disentangling interoception: insights from focal strokes affecting the perception of external and internal milieus. Front. Psychol..

[14] Ferri F, Ardizzi M, Ambrosecchia M, Gallese V (2013). Closing the gap between the inside and the outside: interoceptive sensitivity and social distances. PLoS One.

[15] Critchley HD, Mathias CJ, Dolan RJ (2002). Fear conditioning in humans: the influence of awareness and autonomic arousal on functional neuroanatomy. Neuron.

[16] Azanon E, Longo MR, Soto-Faraco S, Haggard P (2010). The posterior parietal cortex remaps touch into external space. Curr. biology: CB.

[17] Azanon E, Soto-Faraco S (2008). Changing reference frames during the encoding of tactile events. Curr. biology: CB.

[18] Mazzola L, Isnard J, Peyron R, Guenot M, Mauguiere F (2009). Somatotopic organization of pain responses to direct electrical stimulation of the human insular cortex. Pain..

[19] Ibanez A, Manes F (2012). Contextual social cognition and the behavioral variant of frontotemporal dementia. Neurol..

[20] Craig AD (2003). Interoception: the sense of the physiological condition of the body. Curr. Opin. Neurobiol..

[21] Garfinkel SN, Seth AK, Barrett AB, Suzuki K, Critchley HD (2015). Knowing your own heart: distinguishing interoceptive accuracy from interoceptive awareness. Biol. Psychol..

[22] Hassanpour Mahlega S., Yan Lirong, Wang Danny J. J., Lapidus Rachel C., Arevian Armen C., Simmons W. Kyle, Feusner Jamie D., Khalsa Sahib S. (2016). How the heart speaks to the brain: neural activity during cardiorespiratory interoceptive stimulation. Philosophical Transactions of the Royal Society B: Biological Sciences.

[23] Brener Jasper, Ring Christopher (2016). Towards a psychophysics of interoceptive processes: the measurement of heartbeat detection. Philosophical Transactions of the Royal Society B: Biological Sciences.

[24] Ring C, Brener J, Knapp K, Mailloux J (2015). Effects of heartbeat feedback on beliefs about heart rate and heartbeat counting: a cautionary tale about interoceptive awareness. Biol. Psychol..

[25] Kleckner IR, Wormwood JB, Simmons WK, Barrett LF, Quigley KS (2015). Methodological recommendations for a heartbeat detection-based measure of interoceptive sensitivity. Psychophysiol..

[26] Critchley HD (2004). The human cortex responds to an interoceptive challenge. Proc. Natl Acad. Sci. USA.

[27] Duthey B (2013). Priority medicines for Europe and the World-2013 update. Background paper 6-priority diseases and reasons for inclusion. WHO Collaborating Cent. Pharm. Policy Regul..

[28] Murray CJ (2012). Disability-adjusted life years (DALYs) for 291 diseases and injuries in 21 regions, 1990-2010: a systematic analysis for the Global Burden of Disease Study 2010. Lancet.

[29] Schmidt-Wilcke T (2006). Affective components and intensity of pain correlate with structural differences in gray matter in chronic back pain patients. Pain..

[30] Baliki MN (2012). Corticostriatal functional connectivity predicts transition to chronic back pain. Nat. Neurosci..

[31] Apkarian AV, Hashmi JA, Baliki MN (2011). Pain and the brain: specificity and plasticity of the brain in clinical chronic pain. Pain..

[32] Malinen S (2010). Aberrant temporal and spatial brain activity during rest in patients with chronic pain. Proc. Natl Acad. Sci. USA.

[33] Foss JM, Apkarian AV, Chialvo DR (2006). Dynamics of pain: fractal dimension of temporal variability of spontaneous pain differentiates between pain States. J. Neurophysiol..

[34] Iannetti GD, Mouraux A (2010). From the neuromatrix to the pain matrix (and back). Exp. Brain Res..

[35] D’Alessandro G, Cerritelli F, Cortelli P (2016). Sensitization and Interoception as Key Neurological Concepts in Osteopathy and Other Manual Medicines. Front. Neurosci..

[36] Cathcart E, McSweeney T, Johnston R, Young H, Edwards DJ (2019). Immediate biomechanical, systemic, and interoceptive effects of myofascial release on the thoracic spine: A randomised controlled trial. J. Bodyw. Mov. Ther..

[37] Edwards DJ, Young H, Johnston R (2018). The Immediate Effect of Therapeutic Touch and Deep Touch Pressure on Range of Motion, Interoceptive Accuracy and Heart Rate Variability: A Randomized Controlled Trial With Moderation Analysis. Front. Integr. Neurosci..

[38] McGlone F, Cerritelli F, Walker S, Esteves J (2017). The role of gentle touch in perinatal osteopathic manual therapy. Neurosci. Biobehav. Rev..

[39] Wiebking C (2014). GABA in the insula - a predictor of the neural response to interoceptive awareness. Neuroimage.

[40] Critchley HD, Garfinkel SN (2017). Interoception and emotion. Curr. Opin. Psychol..

[41] Critchley HD, Wiens S, Rotshtein P, Ohman A, Dolan RJ (2004). Neural systems supporting interoceptive awareness. Nat. Neurosci..

[42] Chong JSX, Ng GJP, Lee SC, Zhou J (2017). Salience network connectivity in the insula is associated with individual differences in interoceptive accuracy. Brain Struct. Funct..

[43] Corbetta M, Patel G, Shulman GL (2008). The reorienting system of the human brain: from environment to theory of mind. Neuron.

[44] Japee S, Holiday K, Satyshur MD, Mukai I, Ungerleider LG (2015). A role of right middle frontal gyrus in reorienting of attention: a case study. Front. Syst. Neurosci..

[45] Bullmore E, Sporns O (2009). Complex brain networks: graph theoretical analysis of structural and functional systems. Nat. Rev. Neurosci..

[46] He Y, Evans A (2010). Graph theoretical modeling of brain connectivity. Curr. Opin. Neurol..

[47] Bullmore ET, Bassett DS (2011). Brain graphs: graphical models of the human brain connectome. Annu. Rev. Clin. Psychol..

[48] Friston KJ, Harrison L, Penny W (2003). Dynamic causal modelling. Neuroimage.

[49] Cerritelli F, Chiacchiaretta P, Gambi F, Ferretti A (2017). Effect of Continuous Touch on Brain Functional Connectivity Is Modified by the Operator’s Tactile Attention. Front. Hum. Neurosci..

[50] Airaksinen O (2006). Chapter 4. European guidelines for the management of chronic nonspecific low back pain. Eur. spine journal: Off. Publ. Eur. Spine Soc., Eur. Spinal Deformity Society, Eur. Sect. Cerv. Spine Res. Society.

[51] Waddell G (1987). 1987 Volvo award in clinical sciences. A new clinical model for the treatment of low-back pain. Spine.

[52] Spielberger, C. D., Gorsuch, R. L., Lushene, R., Vagg, P. & Jacobs, G. *Manual for the state-trait anxiety inventory (form Y): self-evaluation questionnaire*. (Consulting Psychologists Press Palo Alto, CA, 1983).

[53] Shields SAMMA, Simon A (1989). The Body Awareness Questionnaire: reliability and validity. J. Pers. Assess..

[54] Mehling WE (2009). Body awareness: construct and self-report measures. PLoS One.

[55] Roland M, Fairbank J (2000). The Roland-Morris Disability Questionnaire and the Oswestry Disability Questionnaire. Spine.

[56] Fairbank JC, Couper J, Davies JB, O’Brien JP (1980). The Oswestry low back pain disability questionnaire. Physiotherapy.

[57] Melzack R (1987). The short-form McGill Pain Questionnaire. Pain..

[58] Melzack R (1975). The McGill Pain Questionnaire: major properties and scoring methods. Pain..

[59] Melzack R (2005). The McGill pain questionnaire: from description to measurement. Anesthesiology.

[60] Cicchitti L, Martelli M, Cerritelli F (2015). Chronic inflammatory disease and osteopathy: a systematic review. PLoS One.

[61] Ward, R. *Foundations for Osteopathic Medicine, 2nd edition*. (Lippincott Williams & Wilkins, 1997).

[62] Cerritelli F (2014). Introducing an osteopathic approach into neonatology ward: the NE-O model. Chiropr. Man. Ther..

[63] Pollatos O, Gramann K, Schandry R (2007). Neural systems connecting interoceptive awareness and feelings. Hum. Brain Mapp..

[64] Pollatos O, Schandry R, Auer DP, Kaufmann C (2007). Brain structures mediating cardiovascular arousal and interoceptive awareness. Brain Res..

[65] Wiebking C (2010). Abnormal body perception and neural activity in the insula in depression: an fMRI study of the depressed “material me”. world J. Biol. psychiatry: Off. J. World Federation Societies Biol. Psychiatry.

[66] Schandry R (1981). Heart beat perception and emotional experience. Psychophysiol..

[67] Greicius MD (2007). Resting-state functional connectivity in major depression: abnormally increased contributions from subgenual cingulate cortex and thalamus. Biol. Psychiatry.

[68] Desmond JE, Glover GH (2002). Estimating sample size in functional MRI (fMRI) neuroimaging studies: statistical power analyses. J. Neurosci. Methods.

[69] Chen G, Adleman NE, Saad ZS, Leibenluft E, Cox RW (2014). Applications of multivariate modeling to neuroimaging group analysis: a comprehensive alternative to univariate general linear model. Neuroimage.

[70] Kriegeskorte N, Simmons WK, Bellgowan PS, Baker CI (2009). Circular analysis in systems neuroscience: the dangers of double dipping. Nat. Neurosci..

[71] Modinos G, Ormel J, Aleman A (2009). Activation of anterior insula during self-reflection. PLoS One.

[72] Goswami R, Frances MF, Shoemaker JK (2011). Representation of somatosensory inputs within the cortical autonomic network. Neuroimage.

[73] Decety J, Chen C, Harenski C, Kiehl KA (2013). An fMRI study of affective perspective taking in individuals with psychopathy: imagining another in pain does not evoke empathy. Front. Hum. Neurosci..

[74] Silvers JA, Wager TD, Weber J, Ochsner KN (2015). The neural bases of uninstructed negative emotion modulation. Soc. Cogn. Affect. Neurosci..

[75] Hashmi JA (2013). Shape shifting pain: chronification of back pain shifts brain representation from nociceptive to emotional circuits. Brain.

[76] Simos PG (2017). Neural foundations of overt and covert actions. Neuroimage.

