# Supplementary material for: Effect of manual approaches with osteopathic modality on brain correlates of interoception: an fMRI study
Source: Sci Rep. 2020 Feb 21;10:3214. doi: 10.1038/s41598-020-60253-6 (PMC7035282; doi:10.1038/s41598-020-60253-6)
Supplement: Supplementary file 2 — Research protocol. [file 41598_2020_60253_MOESM2_ESM.docx]

# Research protocol

**Effects of osteopathic manual treatment on interoception: 2- armed RCT on cLBP patients**

ID: InterOst

Researchers
Cerritelli F, Ferretti A, Chiacchiaretta P, Gambi F, Bellomo RG, Barassi G, Saggini R, Romani GL

Research centres:

Department of Neuroscience and Imaging
Institute for Advanced Biomedical Technologies, ‘‘G. d’Annunzio University’’ Foundation, Chieti, Italy

Interoception is described as the perceived sense of the physiological condition of the body [1] which is distinguished from exteroception that is characterized by the code of information deriving from somatic motor activity [2]. The first representation of the interoceptive information is on the posterior-dorsal insula conrtex [2] and subsequently integrated into the right anterior insula [2, 3], hence the information is projected towards the amygdala and the nucleus accumbens [ 4] and towards the orbito-frontal cortex [2, 5]. Therefore, the insula is strategically positioned to receive and integrate interoceptive information [6] and exteroceptive and integrate them with emotions and memory giving the perception of the self and how the self is perceived [7, 8]. In addition, data from other studies extended the role of multimodal convergence of the insula. Indeed, it seems to be involved in gustatory responses, homeostatic processes, in vestibular function, attention, pain, emotion, verbal and motor information, as well as on olfactory, visual, auditory and tactile sensations [1-3, 9]. This functional feature of convergence of numerous and different information lays the foundations for a cinemascopic representation model of perceptions of self argued by Craig as "sentient self" [8] that underlies the insula's ability to generate global emotional moments over time, second by second [8, 10]. Moreover, the insula seems to be the central hub of the "salience network" (SN), which includes the dorsolateral prefrontal cortex and the anterior insula, in the complex called fronto-insular cortex [11] and acts as a mediator of information in the cognitive representation of self. This SN is one of the two "functional connectivity" circuits (FC) to which the insula participates. The first, anterior, has as its distinctive elements the ventral anterior insula, the rostral anterior cingulate cortex, the inferior and medial frontal cortex, and the temporoparietal cortex. The second, instead, is characterized by the presence of the posterior dorsal insula, the posterior-dorsal cingulate cortex, the sensorimotor, premotor, supplementary motor, the temporal cortex and some occipital areas. The two circuits serve two different functions: the first to elaborate the interoceptive information and control the patterns of attentional processing, therefore mainly involved in the functions of cognitive integration, homeostatic, emotional, interoceptive awareness and control of the arousal during cognitive tasks. [12-14]; the second control of motor function, environmental and selective response [2, 15, 16].

The correlations between the various brain areas appear to be exclusive. In fact, the individual structures of each network seem to be anti-correlated. This reinforces the idea that the two portions of the insula underlie different functions and are connected to different networks that work independently [17].

One way to measure the functional connections between the various brain areas is during the "resting state" condition, in which the subject is in the absence of stimuli. Fluctuations of brain activity are recorded using fMRI with BOLD technique [18]. This allows to highlight spontaneous activity patterns known as resting state networks (RSN) [13, 17, 19, 20]. The properties of the BOLD signal of the RSN seem to be consistent between individuals and stable between different measurement sessions [21-23]. The SN and therefore the insula circuits have also been studied in the field of RSN, with more precise areas of brain involvement [11, 12]. Menon and Uddin have hypothesized that the right anterior insula acts as a causal center of efference that coordinates two important networks of executive (external, posterior insula) and interoceptive (internal, anterior insula) control [11]. Thus the connection hypotheses formulated by Craig [1-3, 24] as well as the SN [11, 12] define the role of the right insular cortex as a pivotal region in the brain's attentive system [25, 26].

Moreover, some authors have studied the activation of the insula cortex in relation to specific tasks: interoceptive (heartbeat [27] or breath [28]) and exteroceptive [29], showing significantly different behaviors.

Chronic pain is one of the most frequent chronic disorders and has the highest prevalence in people with low back pain (cLBP), which is the main cause of economic and financial loss in terms of cost per treatment and days of work lost [30]. In Europe, the prevalence of cLBP varies from country to country. In Italy the prevalence of cLBP is approximately 6% [31]. While there is a high level of knowledge of the peripheral and spinal neurological mechanisms of chronic pain, evidence about central changes is emerging [32]. Numerous studies on the central mechanisms of chronic pain refer to neurogenic (or neuropathic) pain [33] which appears to be related to thalamocortical patterns measured both by EEG [34, 35] and by fMRI [3, 36, 37]. Furthermore, chronic pain seems to be related to specific cerebral fluctuations [38]. Evidence suggests that different brain structures are responsible of central changes in subjects with chronic pain [39], in particular the anterior circuit of the insula thus the anterior insula and the anterior cortex of the cingulum [40]. However, authors suggested the predominance of the medial prefrontal cortex [41]. Moreover, Apkarian et al (2011) estimated that by analyzing the activity of the medial prefrontal cortex and insula it could be estimated the magnitude and duration of lumbar pain with a margin of error of 20% [41]. Furthermore, Malinen et al showed that subjects with chronic pain have a condition of activation of abnormal RSN, with a higher activity frequency on insula and on the anterior cingulate cortex [42]. Numerous types of interdisciplinary treatments, including manipulative therapies, have been proposed to improve clinical outcomes in patients with cLBP [43]. However, there is still little evidence of the possibility of changes in brain activity in subjects with chronic pain using manual therapies, for example with an osteopathic approach. The osteopathic model uses a system of manual evaluation of the patient, defined osteopathic analysis, in order to highlight the presence of somatic dysfunctions (codes M99.00-09 classes ICD10), that is body districts with mobility restriction. Osteopathy addresses somatic dysfunctions through appropriate manual methods (OMT), with the aim of normalizing them [44]. The manual analysis with osteopathic modality is addressed to the analysis of body movements in order to highlight the mobility limitations of the single joints. The objective of this study is to evaluate the effect of osteopathic treatment on functional connectivity and on insula activity and therefore of the anterior circuit of the SN, both in resting state and during an 'interoceptive awareness' - IA - (heart rate) or 'exteroceptive awareness' - EA - (time count) task in a sample of subjects suffering from cLBP. This could shed light on the central effect of manual treatment with osteopathic modality, laying the first bases for understanding neural mechanisms.

MATERIALS AND METHODS

PURPOSE OF THE STUDY

The main aim of the present randomized controlled two-arm and single-blind study is to evaluate the change in activation and connectivity of the anterior cortex of the insula and the medial prefrontal cortex between before and after the period of manual treatment with osteopathic modality. Secondary goals are:

I) to monitor the differences of activity and FC of the SN between the study group and the sham control;

II) to monitor the intra-group differences and the intergroup of activities and of the CF of the SN immediately after the first treatment;

III) to monitor the differences in activity of intra-group and inter-group RSN during the treatment period;

IV) to monitor the differences in the IA and EA both within and between groups;

V) to evaluate the DTI structural differences;

VI) to evaluate differences in terms of GABA concentration in the anterior insula by means of

MRS

**POPULATION STUDY**

*Study population*

The reference population of the study will consist of men and women aged between 30 and 50, suffering from chronic low back pain with a minimum period of pain of 6 months. Evaluation will follow the criteria published by Treede et al and the International Association for the Study of Pain (IASP) [33].

The exclusion criteria are shown in table 1.

Subjects who give their consent to participate in the study will be enrolled and randomly assigned to the study group or sham.

*Recruitment*

All the subjects belonging to the Physical and Rehabilitative Medicine Sector of the university of sports medicine (CUMS) of Chieti, will be recruited by an expert MD who will decide to include or exclude the participant from the study.

**STUDY / CONTROL GROUP**

*Randomization*

All patients included in the study are registered with a progressive identifier (patient code). The treatment arm will be assigned by random selection procedure. A computerized randomization algorithm based on the binomial distribution constructs a balanced and progressive list of random values ​​assigned within a specific interval (in this case 1 = group A, 0 = group B), this is also called randomization code. A progressive index is associated with each value. Treatment is assigned by assigning to each patient the randomization code relative to the equivalent patient code.

The randomization process will be based on the use of a software for randomization (R statistical software) [45] and conducted by a "permuted block" ratio of 1: 1.

The study foresees two groups (one of study and one of control) of 20 subjects each (= figure 1). The study group (OMT) will consist of 20 subjects who will receive osteopathic manipulative treatment. The sham control group will receive a similar touch without any technique.

**TYPE OF INTERVENTION**

*Study group*

Patients randomized in the study group will be treated with osteopathic modality; treatments will be performed with the following schedule: a weekly session for 4 treatments for a duration of 30 minutes. The treatments will be administered by 1 expert osteopath. The techniques used will be those of ligamentous-membranous-Guidance balancing, in line with the principles available in the literature. The treatments will be carried out at the Physical and Rehabilitative Medicine Sector of the University of Sports Medicine (CUMS) in Chieti.

*Sham control group*

Patients randomized in the sham group will receive treatment with the same timing and methodology applied to the study group. The treatments will be carried out by 1 healthcare professional with the same background. The sessions will be carried out at the Physical and Rehabilitative Medicine Sector of the University of Sports Medicine (CUMS) of Chieti.

All subjects will be asked during the treatment period (4 weeks) to avoid the use of drugs.

*Patient preparation*

Subjects will be instructed to simply keep their eyes closed, not to think about anything in particular and not fall asleep. After the session of the measurement session, the participants will be asked if they have fallen asleep during the recording, and the data of the subjects with positive and doubtful answer will be excluded from the study.

**BLINDING / MASKING**

Randomization will be performed by the Department of Neuroscience and Imaging of the University of Chieti, which will take care of the control of all processes.

Staff blinding will be respected through the following principles: only one MD will perform the initial and final evaluation of the subjects included in the study. Operators who perform measurements with fMRI will not be aware of the subjects in the sham study and control group. None of the operators involved will be aware of the purpose and design of the study.

Subjects included in the study will not be aware of the group to which they belong considering the inclusion criteria related to osteopathic treatment.

The blinding of osteopathic operators and sham operators can not be maintained due to the active nature of the treatment.

**OUTCOMES**

*Functional Magnetic Resonance (fMRI)*

Acquisitions will be performed with a 3T MR tomograph at ITAB. The following recordings will be made with fMRI:

1) baseline

2) immediately after the first session

3) After 1 month, or at the end of the treatment period

The sequences that will be used are:

1) Eco-planar weighs in T2 * for fMRI measurements with contrast Blood Oxygenation

Level Dependent (BOLD) and Pseudo-continuous Arterial Spin Labeling (pCASL) for the Brain blood flow (CBF) measurements

2) Diffusion tensor imaging (DTI) sequences for the study of structural connectivity.

3) Spectroscopy sequences for the measurement of GABA concentrations.

The anatomical target will be the anterior insula.

*fMRI task*

A standard method to evaluate interoceptive and exteroceptive awareness will be used. This method is based on a paradigm already experimented in previous studies [27, 29, 46, 47]. This consists of three independent conditions presented in causal order for 15-20 sec each. The three conditions are: a task for IA, one for EA and a resting state period. The task of AI is to count own heart beats. EA's task is to count the rhythm of a sound stimulus, a piece of music, received in headphone. The period of fixation consists of voluntarily reducing cognitive activities to a minimum.

All the stimuli will be distinguished by a specific symbol on the display, which will also control the time for each individual task. When the task is finished, the subjects will report on a "visual analogue scale" the number of heart beats or rhythm deriving from the task.

*Diffusion Tensor Imaging (DTI)*

Structural connectivity will be investigated using the DTI, focusing on possible changes after the treatment period.

*Timing of instrumental acquisitions*

The instrumental acquisitions will last 50 minutes and will be divided as follows:

1) 5 'anatomical acquisition in T1

2) 10 'MRS

3) 10 'BOLD resting state

4) 5 'pCASL

5) 10 'DTI

6) 10 'BOLD TASK

*Questionnaires*

Patients included in the study will have to answer the following questionnaires in relation to the various times:

At inclusion

• General questionnaire with the purpose of collecting information on the social demographic status, drugs used, pain location and previous treatments.

• Temperament Evaluation of the Memphis, Pisa, Paris and San Diego - Autoquestionnaire (TEMPS-A). Self-administered questionnaire that measures temperament.

• Beck Depression Inventory - II (BDI-II), a questionnaire used as an indicator of the presence and intensity of depressive symptoms.

• Clinical scale of anxiety and depression - HADS. Questionnaire for the assessment of anxiety and depression in the non-psychiatric population.

• Short Form 36 version 2 Health Survey (SF-36v2). Generic questionnaire on the quality of life. Composed of 36 questions spread over 8 main domains. Administration occurs at inclusion and after 4 weeks [48].

Pain

• McGill pain questionnaire [49], a specific questionnaire on low back pain.

Disability

• Oswestry Disability and Pain Index [50], specific questionnaire on consequences

of lumbar pain.

• Body Awareness Questionnaire (BAQ), questionnaire to assess self awareness [51]

Next to each treatment

• McGill pain questionnaire [49]

• Oswestry disability and pain index [50]

• BDI-II

• HADS

At the end of the study period

• SF-36v2 [48]

• Satisfaction with treatment [49]

• McGill Questionnaire [49]

• Oswestry disability and pain index [50]

• Body Awareness Questionnaire (BAQ) [51]

• BDI-II

• HADS

Safety will be assessed by monitoring the adverse events reported by the patient at each manual treatment session with osteopathic modality or sham treatment. The acceptance of the treatment will be evaluated through the drop-outs and the completeness of the questions on the satisfaction of the treatment and will try to try again the OMT.

**PROCEDURE FOR DATA COLLECTION AND TREATMENT**

Data collection will be performed using a specific software, developed ad-hoc for the purpose of the study.

The data will be processed according to the privacy law. All information will be encrypted, coded and made anonymous. The acquired data will be used exclusively for the specific purpose of the research.

**ETHICS COMMITTEE, INFORMED CONSENT and REGISTRATION TRIAL**

Eligible subjects will be required to provide informed consent, in accordance with the Helsinki Declaration, to enter the trial. This protocol will be registered on www.clinicaltrial.gov, only after the approval of the ethics committee.

**MRI DATA PROCESSING**

The functional, structural and spectroscopic magnetic resonance data will be analyzed with dedicated software (AFNI, FSL, JMRUI) or specifically developed at ITAB, obtaining both individual and group functional activation and connectivity maps. Differences in activity and connectivity pre / post or between groups in the insula and other regions of interest will be evaluated through linear mixed effect models.

**STATISTIC ANALYSIS**

The lack of literature on precise estimate of the effect (effect size) of the manual treatment with osteopathic modality on the main outcome limits the calculation of the sample size. Therefore, given the lack of research measuring these effects at the brain level using fMRI, it is rather difficult to have a precise effect-size estimation. However, in general, the estimates reported in the literature for the expected effect-size with fMRI measures (eg comparison of connectivity values between different populations as in Greicius et al., 2007) indicate relatively high values (Cohen ‘s d= 1.01). This effect-size, together with an alpha and beta value of 0.05 and 0.8 respectively as usual in neuroimaging studies (Desmond et al., 2002) have been included in the R Statistical program to estimate the sample size, resulting in N=16 subjects per group.

Statistical analysis will be based on the 'intention-to-treat' and 'per-protocol' method. The statistical analysis will use the mean and standard deviation, median and percentiles, percentage points for the description of the variables of the two groups. The analyzes will be standardized by age and gender. The hypothesis H0 (null hypothesis) is the non-difference between the two groups at the end of the treatment. To test this hypothesis the assumption of the normal distribution of data through the Shapiro test will be tested first.

The analysis of residuals indicates the possible use of logarithmic transformations of the data. Subsequently the univariate analysis foresees the use of the analysis of the variance (ANOVA) for the comparison of the numerical variables, while the chi square (chi-square test) for the categorical and / or ordinal variables. Primarily used for the comparison of the two samples to the baseline, with the same method we will also analyze the data at the end of the treatment taking into account the inability to detect the independent effect of the variables on the outcome. ANCOVA will be used to explore in greater detail the treatment effect considering cofactors. Multivariate analysis will include the use of the best weighed model for confounding and interaction factors.

The level of statistical significance is established for an alpha <0.05. A post-hoc power calculation will be carried out.

Table 1. Exclusion criteria

• Subjects in acute phase,

• Subjects aged under 30 and over 50

• Associated co-morbidities (i.e. metabolic syndrome)

• Subjects with previous surgical operations,

• Subjects with congenital anomalies,

• Cardio-vascular, respiratory, gastro-enteric diseases,

• Pregnant women,

• Subjects with chronic pain> 10 years

• Subjects with psychiatric illness or overt psychic disorders

• Subjects with vertigo syndrome

• Subjects with associated cervicalgia

• Obese subjects

• Diabetic subjects

• Subjects in pharmacology

• Subjects without a history of drug or alcohol abuse

• Subjects under physiotherapy

• Subjects with experience of manual treatments with osteopathic modality

**Bibliography**

1. Craig, A.D., *Interoception: the sense of the physiological condition of the body.* Curr Opin Neurobiol, 2003. **13**(4): p. 500-5.
2. Craig, A.D., *How do you feel? Interoception: the sense of the physiological condition of the body.* Nat Rev Neurosci, 2002. **3**(8): p. 655-66.
3. Craig, A.D., *How do you feel--now? The anterior insula and human awareness.* Nat Rev Neurosci, 2009. **10**(1): p. 59-70.
4. Reynolds, S.M. and D.S. Zahm, *Speci6city in the projections of prefrontal and insular cortex to ventral striatopallidum and the extended amygdala.* J Neurosci, 2005. **25**(50): p. 11757-67.
5. Ongur, D. and J.L. Price, *The organization of networks within the orbital and medial prefrontal cortex of rats, monkeys and humans.* Cereb Cortex, 2000. **10**(3): p. 206- 19.
6. Paulus, M.P. and M.B. Stein, *An insular view of anxiety.* Biol Psychiatry, 2006. **60**(4): p. 383-7.
7. Bonthius, D.J., A. Solodkin, and G.W. Van Hoesen, *Pathology of the insular cortex in Alzheimer disease depends on cortical architecture.* J Neuropathol Exp Neurol, 2005. **64**(10): p. 910-22.
8. Craig, A.D., *The sentient self.* Brain Struct Funct, 2010. **214**(5-6): p. 563-77.
9. Nagai, M., K. Kishi, and S. Kato, *Insular cortex and neuropsychiatric disorders: a*

*review of recent literature.* Eur Psychiatry, 2007. **22**(6): p. 387-94.

1. Livesey, A.C., M.B. Wall, and A.T. Smith, *Time perception: manipulation of task*

*dif6culty dissociates clock functions from other cognitive demands.*

Neuropsychologia, 2007. **45**(2): p. 321-31.

1. Menon, V. and L.Q. Uddin, *Saliency, switching, attention and control: a network*

*model of insula function.* Brain Struct Funct, 2010. **214**(5-6): p. 655-67.

1. Seeley, W.W., et al., *Dissociable intrinsic connectivity networks for salience*

*processing and executive control.* J Neurosci, 2007. **27**(9): p. 2349-56.

1. Fox, M.D., et al., *Spontaneous neuronal activity distinguishes human dorsal and*

*ventral attention systems.* Proc Natl Acad Sci U S A, 2006. **103**(26): p. 10046-51.

1. Dosenbach, N.U., et al., *A core system for the implementation of task sets.* Neuron,

2006. **50**(5): p. 799-812.

1. Kurth, F., et al., *Cytoarchitecture and probabilistic maps of the human posterior*

*insular cortex.* Cereb Cortex, 2010. **20**(6): p. 1448-61.

1. Kurth, F., et al., *A link between the systems: functional differentiation and integration*

*within the human insula revealed by meta-analysis.* Brain Struct Funct, 2010. **214**(5-

6): p. 519-34.

1. Cauda, F., et al., *Functional connectivity of the insula in the resting brain.*

Neuroimage, 2011. **55**(1): p. 8-23.

1. Fox, M.D. and M.E. Raichle, *Spontaneous :uctuations in brain activity observed*

*with functional magnetic resonance imaging.* Nat Rev Neurosci, 2007. **8**(9): p. 700-

11.

1. Vincent, J.L., et al., *Intrinsic functional architecture in the anaesthetized monkey*

*brain.* Nature, 2007. **447**(7140): p. 83-6.

1. De Luca, M., et al., *fMRI resting state networks de6ne distinct modes of long-*

*distance interactions in the human brain.* Neuroimage, 2006. **29**(4): p. 1359-67.

1. Damoiseaux, J.S., et al., *Consistent resting-state networks across healthy subjects.*

Proc Natl Acad Sci U S A, 2006. **103**(37): p. 13848-53.

1. Zuo, X.N., et al., *The oscillating brain: complex and reliable.* Neuroimage, 2010.

**49**(2): p. 1432-45.

1. Shehzad, Z., et al., *The resting brain: unconstrained yet reliable.* Cereb Cortex,

2009. **19**(10): p. 2209-29.

1. Craig, A.D., *Human feelings: why are some more aware than others?* Trends Cogn

Sci, 2004. **8**(6): p. 239-41.

1. Nelson, S.M., et al., *Role of the anterior insula in task-level control and focal*

*attention.* Brain Struct Funct, 2010. **214**(5-6): p. 669-80.

1. Sridharan, D., D.J. Levitin, and V. Menon, *A critical role for the right fronto-insular*

*cortex in switching between central-executive and default-mode networks.* Proc Natl Acad Sci U S A, 2008. **105**(34): p. 12569-74.

11

1. Pollatos, O., et al., *Brain structures mediating cardiovascular arousal and interoceptive awareness.* Brain Res, 2007. **1141**: p. 178-87.
2. Farb, N.A., Z.V. Segal, and A.K. Anderson, *Attentional modulation of primary interoceptive and exteroceptive cortices.* Cereb Cortex, 2013. **23**(1): p. 114-26.
3. Wiebking, C., et al., *GABA in the insula - a predictor of the neural response to interoceptive awareness.* Neuroimage, 2013.
4. Ricci, J.A., et al., *Back pain exacerbations and lost productive time costs in United States workers.* Spine (Phila Pa 1976), 2006. **31**(26): p. 3052-60.
5. Airaksinen, O., et al., *Chapter 4. European guidelines for the management of chronic nonspeci6c low back pain.* Eur Spine J, 2006. **15 Suppl 2**: p. S192-300.
6. Lee, M.C. and I. Tracey, *Unravelling the mystery of pain, suffering, and relief with brain imaging.* Curr Pain Headache Rep, 2010. **14**(2): p. 124-31.
7. Treede, R.D., et al., *Neuropathic pain: rede6nition and a grading system for clinical and research purposes.* Neurology, 2008. **70**(18): p. 1630-5.
8. Sarnthein, J., et al., *Increased EEG power and slowed dominant frequency in patients with neurogenic pain.* Brain, 2006. **129**(Pt 1): p. 55-64.
9. Schmidt, S., et al., *Pain ratings, psychological functioning and quantitative EEG in a controlled study of chronic back pain patients.* PLoS One, 2012. **7**(3): p. e31138.
10. Iannetti, G.D. and A. Mouraux, *From the neuromatrix to the pain matrix (and back).* Exp Brain Res, 2010. **205**(1): p. 1-12.
11. Craig, A.D., *A new view of pain as a homeostatic emotion.* Trends Neurosci, 2003. **26**(6): p. 303-7.
12. Foss, J.M., A.V. Apkarian, and D.R. Chialvo, *Dynamics of pain: fractal dimension of temporal variability of spontaneous pain differentiates between pain States.* J Neurophysiol, 2006. **95**(2): p. 730-6.
13. Schmidt-Wilcke, T., et al., *Affective components and intensity of pain correlate with structural differences in gray matter in chronic back pain patients.* Pain, 2006. **125**(1-2): p. 89-97.
14. Baliki, M.N., et al., *Corticostriatal functional connectivity predicts transition to chronic back pain.* Nat Neurosci, 2012. **15**(8): p. 1117-9.
15. Apkarian, A.V., J.A. Hashmi, and M.N. Baliki, *Pain and the brain: speci6city and plasticity of the brain in clinical chronic pain.* Pain, 2011. **152**(3 Suppl): p. S49-64.
16. Malinen, S., et al., *Aberrant temporal and spatial brain activity during rest in patients with chronic pain.* Proc Natl Acad Sci U S A, 2010. **107**(14): p. 6493-7.
17. Rubinstein, S.M., et al., *Spinal manipulative therapy for chronic low-back pain.* Cochrane Database Syst Rev, 2011(2): p. CD008112.
18. R. C. Ward, et al., *Foundations for Osteopathic Medicine*. 2nd Revised edition ed2002: Lippincott Williams & Wilkins.
19. R Development Core Team, *R: A language and environment for statistical computing.*, in *R Foundation for Statistical Computing*2012: Vienna, Austria.
20. Pollatos, O., K. Gramann, and R. Schandry, *Neural systems connecting interoceptive awareness and feelings.* Hum Brain Mapp, 2007. **28**(1): p. 9-18.
21. Wiebking, C., et al., *Abnormal body perception and neural activity in the insula in depression: an fMRI study of the depressed "material me".* World J Biol Psychiatry, 2010. **11**(3): p. 538-49.
22. McHorney, C.A., et al., *The MOS 36-item Short-Form Health Survey (SF-36): III. Tests of data quality, scaling assumptions, and reliability across diverse patient groups.* Med Care, 1994. **32**(1): p. 40-66.
23. Melzack, R., *The short-form McGill Pain Questionnaire.* Pain, 1987. **30**(2): p. 191-7.
24. Fairbank, J.C., et al., *The Oswestry low back pain disability questionnaire.*

Physiotherapy, 1980. **66**(8): p. 271-3.

1. Shields, S.A.M., M.A. Simon, A., *The Body Awareness Questionnaire: reliability and*

*validity.* J Pers Assess, 1989. **53**: p. 802:815.
